# Exploratory Outlier Detection for Acceleromyographic Neuromuscular Monitoring: Machine Learning Approach

**DOI:** 10.2196/25913

**Published:** 2021-06-21

**Authors:** Michaël Verdonck, Hugo Carvalho, Johan Berghmans, Patrice Forget, Jan Poelaert

**Affiliations:** 1 Department of Anesthesiology and Perioperative Medicine Vrije Universiteit Brussel Jette Belgium; 2 Department of Anesthesiology and Perioperative Medicine Universitair Ziekenhuis Brussel Brussel Belgium; 3 Department of Anesthesiology Ziekenhuis Netwerk Antwerpen Antwerp Belgium; 4 Department of Anaesthesia University of Aberdeen Aberdeen United Kingdom

**Keywords:** neuromuscular monitoring, outlier analysis, acceleromyography, postoperative residual curarization, train-of-four, monitoring devices, neuromuscular, machine learning, monitors, anesthesiology

## Abstract

**Background:**

Perioperative quantitative monitoring of neuromuscular function in patients receiving neuromuscular blockers has become internationally recognized as an absolute and core necessity in modern anesthesia care. Because of their kinetic nature, artifactual recordings of acceleromyography-based neuromuscular monitoring devices are not unusual. These generate a great deal of cynicism among anesthesiologists, constituting an obstacle toward their widespread adoption. Through outlier analysis techniques, monitoring devices can learn to detect and flag signal abnormalities. Outlier analysis (or anomaly detection) refers to the problem of finding patterns in data that do not conform to expected behavior.

**Objective:**

This study was motivated by the development of a smartphone app intended for neuromuscular monitoring based on combined accelerometric and angular hand movement data. During the paired comparison stage of this app against existing acceleromyography monitoring devices, it was noted that the results from both devices did not always concur. This study aims to engineer a set of features that enable the detection of outliers in the form of erroneous train-of-four (TOF) measurements from an acceleromyographic-based device. These features are tested for their potential in the detection of erroneous TOF measurements by developing an outlier detection algorithm.

**Methods:**

A data set encompassing 533 high-sensitivity TOF measurements from 35 patients was created based on a multicentric open label trial of a purpose-built accelero- and gyroscopic-based neuromuscular monitoring app. A basic set of features was extracted based on raw data while a second set of features was purpose engineered based on TOF pattern characteristics. Two cost-sensitive logistic regression (CSLR) models were deployed to evaluate the performance of these features. The final output of the developed models was a binary classification, indicating if a TOF measurement was an outlier or not.

**Results:**

A total of 7 basic features were extracted based on raw data, while another 8 features were engineered based on TOF pattern characteristics. The model training and testing were based on separate data sets: one with 319 measurements (18 outliers) and a second with 214 measurements (12 outliers). The F1 score (95% CI) was 0.86 (0.48-0.97) for the CSLR model with engineered features, significantly larger than the CSLR model with the basic features (0.29 [0.17-0.53]; *P*<.001).

**Conclusions:**

The set of engineered features and their corresponding incorporation in an outlier detection algorithm have the potential to increase overall neuromuscular monitoring data consistency. Integrating outlier flagging algorithms within neuromuscular monitors could potentially reduce overall acceleromyography-based reliability issues.

**Trial Registration:**

ClinicalTrials.gov NCT03605225; https://clinicaltrials.gov/ct2/show/NCT03605225

## Introduction

Postoperative residual curarization remains a frequent and often concealed event within modern anesthesia care [1]. It translates clinically into complications such as aspiration of gastric contents [2,3] and an impaired ventilatory response to hypoxia [4]. This is ultimately linked to an increase of morbidity and mortality due to postoperative pulmonary complications [5]. As such, perioperative quantitative monitoring of neuromuscular function in patients receiving neuromuscular blockers has become internationally recognized as an absolute and core necessity in modern anesthesia care [6,7]. Besides a reduction of the incidence of severe respiratory complications [8-10], quantitative monitoring also potentially leads to considerable financial health care savings, with complications stemming from suboptimal neuromuscular monitoring being estimated to be as high as US $25.000 per patient per event [7].

Although a seemingly straightforward procedure, neuromuscular monitoring presents users with nuances that are frequently overlooked or that are prone to misinterpretation [11]. This has been exemplified by research [12,13] showing that baseline (control) train-of-four ratios (TOFRs; T4/T1) at the adductor pollicis frequently assume supra-physiological values (TOFR > 1) when measured using acceleromyography (AMG). Similarly, Kopman et al [13] have scrutinized some algorithmic simplifications used by common AMG monitors (T4/T2 ratio as a substitute for T4/T1) and how their validity is dependent on the degree of recovery from nondepolarizing neuromuscular block. Such interpretative considerations, associated frequent artifactual confounders, and known overestimation tendencies when compared with electromyography (EMG) or mechanomyography [14] contribute to the perpetuation of anesthesiologist’s cynicism toward objective neuromuscular monitoring methods, further hindering their widespread adoption [15].

The herein presented research has been motivated by the development of a smartphone app intended for neuromuscular monitoring based on combined accelerometric and angular hand movement data [16]. During the paired comparison stage of this app against existing AMG monitoring devices, it was noted that the results from both devices did not always concur. For instance, it was observed that the collected raw movement data regularly displayed nonstandard TOF patterns, whereas the AMG neuromuscular monitoring device did not appear to detect these outliers and displayed a seemingly (oversimplified) TOFR plotting. As with any instrument that aims to measure a certain signal [17], the measurement of TOFR is similarly prone to the appearance of outliers, which can be erroneously interpreted as correct measurements.

From a data analysis standpoint, outlier analysis techniques can be adopted to increase data reliability. Outlier analysis (or anomaly detection) refers to the problem of finding patterns in data that do not conform to expected behavior [18]. This study aims to conduct an offline exploratory analysis on raw AMG neuromuscular monitoring data and to engineer features (or variables) to be able to flag erroneous TOF measurements. These features will be subsequently evaluated for their usability in outlier analysis.

## Methods

### Overview

This manuscript follows the “Guidelines for Developing and Reporting Machine Learning Predictive Models in Biomedical Research: A Multidisciplinary View” [19]. The key steps of the feature engineering and the development of the outlier analysis algorithm are summarized below.

### Data Recruitment and Preprocessing

All data for this study have been collected during a prospective open-label bicentric clinical trial (Clinical Trial Identifier NCT03605225) that took place in Ziekenhuisnetwerk Antwerpen Middelheim (Antwerp, Belgium) and Universitair Ziekenhuis Brussel (Brussels, Belgium). Registration occurred prior to the start of the trial. Data collection started in February 2018 and terminated in April 2019. The trial was conducted in accordance with the established protocol after approval by the Medical Ethical Committees of both hospitals (ZNA Middelheim reference number 5055; UZBrussel reference 2018/031, BUN 009201835039). It followed current good clinical practice guidelines and applicable law(s), as well as adhered to the applicable CONSORT guidelines.

The data used for the algorithm development were collected using a purpose-built smartphone app specifically aimed to monitor hand movements evoked by extraneural supramaximal stimulation of the ipsilateral nervus ulnaris by means of a peripheral nerve stimulator. Collected data included triaxial (3D) raw acceleration values (m/s^2^) as well as raw 3D angular velocity values (rad s^–1^).

Earlier trials involving beta versions of this app have been published and they reported bidirectional 95% limits of agreement of 0.12 (TOFR, absolute units) when compared with a standard AMG neuromuscular CE/FDA-labeled monitor [16]. This study included 35 patients, with a total of 533 TOF measurements. The offline evaluation of the observations was performed by 2 authors of this paper (MV and HC), who performed the evaluation independently from one another. The classification of outliers was afterward compared and corresponded to a 98% agreement on the labeling of TOF patterns. In total, 30 of the 533 observations were identified as outliers. These anomalies were detected in the TOF measurements of 18 patients.

### Feature Engineering

**Figure 1:**
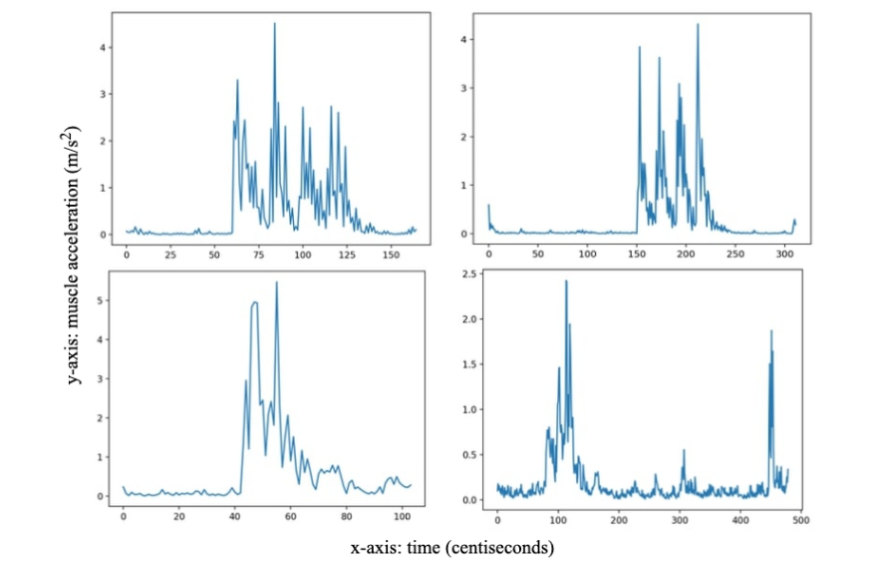
Normal and anomalous train-of-four (TOF) patterns; upper figures display normal TOF observations while bottom figures represent anomalies. Normality recordings are illustrated by the upper 2 patterns, where 4 clear peaks can be detected and that follow one another in a fixed time interval. The lower 2 patterns represent 2 simulated anomalies, where the 4 peaks cannot be clearly recognized from the TOF pattern; or where a wide gap in terms of time interval exists between peaks.

The acceleration and angulation signals were collected through the open-source Cordova Plugin Device-motion library [20], and measure the movement of the muscle contractions in the 3 orthogonal directions of movement (X, Y, and Z). Similar to studies in mechanomyography [21], root-mean square analysis was performed on these signals to indicate the range of muscle displacement represented by its acceleration (expressed with units of m/s^2^; Figure 1). The features for the model were then directly derived from the continuous monitoring signal. A first basic set of features (Table 1) were derived: the combined acceleration value of multiple points on the vicinity of the peak of the different TOF twitches (T1, T2, T3, T4) and the absolute TOFR value.

**Table 1:** Description of the basic features of data set. A train-of-four recording is defined as the integral combination of all acceleration/angulation points of T1, T2, T3, and T4.

Feature name	Description	n (count)
T1	First twitch of train-of-four response	533
T2	Second twitch of train-of-four response	533
T3	Third twitch of train-of-four response	533
T4	Fourth twitch of train-of-four response	533
TOFR^a^	Absolute ratio derived by dividing T1 with T4	533
AMG^b^_StdDev	Standard deviation of an AMG measurement	533
AMG_Mean	Arithmetic mean of an AMG measurement	533

^a^TOFR: train-of-four ratio.

^b^AMG: acceleromyography.

**Figure 2:**
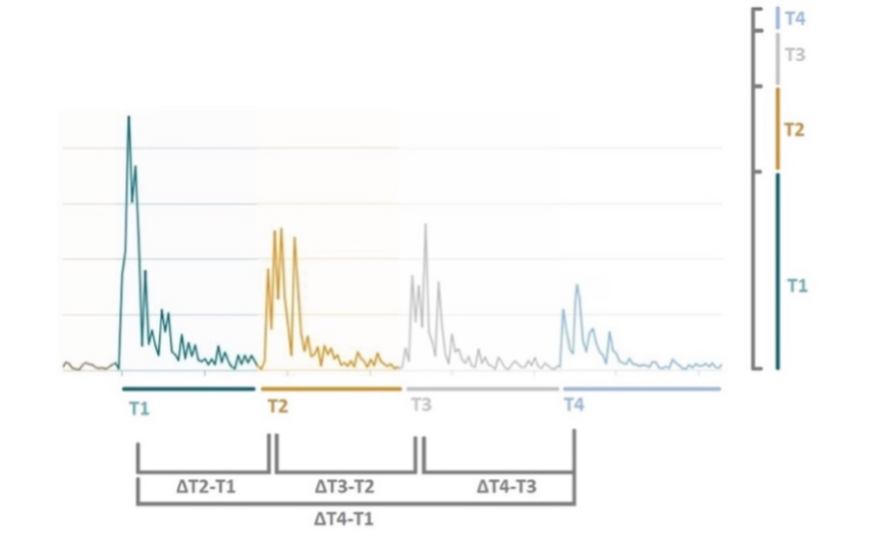
Basic and engineered feature illustration (x axis: time, y axis: combined angulation and acceleration). Each color represents an individual train-of-four (TOF) twitch. Each individual twitch (T1, T2, T3, and T4) is composed of multiple acceleration/angulation points during the corresponding contraction, and not solely by the highest value. The TOF recording is obtained by the summation of each individual twitch.

Additionally, the arithmetic mean and standard deviation of the AMG values related to one measurement were computed in order to gain a better insight into the differences in variation between different AMG measurements. In order to avoid confusion with other descriptive statistics, these were labeled as “AMG_StdDev” and “AMG_Mean.” An additional set of features were engineered to assess specific TOF pattern characteristics (Table 2). Several of these were derived from the distance between the different TOF twitches (denominated with the prefix “delta”; Figure 2), whereas other features were based on the ratio of a specific TOF twitch compared with the mean of the respective TOF measurement (denominated with the prefix “ratio”). With a nonanomalous pattern, one would logically expect such a ratio to be exceedingly higher than the arithmetic mean of the TOF measurement. In total, 15 features were extracted and engineered to serve as input for the outlier detection models. We emphasize that the authors have deliberately designed the aforementioned features to detect an anomaly based on the signal characteristics of a specific measurement instance. Because of the limited size of the data set, there is no feature that takes into account any historical information of the TOF recording, administered drugs, or other patient-related parameters.

**Table 2:** Description of engineered features of data set.

Feature name	Description	n (count)
deltaT2_T1	Elapsed time (ms) between second and first twitch	533
deltaT3_T2	Elapsed time (ms) between third and second twitch	533
deltaT4_T3	Elapsed time (ms) between fourth and third twitch	533
deltaT4_T1	Elapsed time (ms) between fourth and first twitch	533
ratioT1	Ratio between first twitch and arithmetic mean	533
ratioT2	Ratio between second twitch and arithmetic mean	533
ratioT3	Ratio between third twitch and arithmetic mean	533
ratioT4	Ratio between fourth twitch and arithmetic mean	533

### Model Development and Overfitting

**Figure 3:**
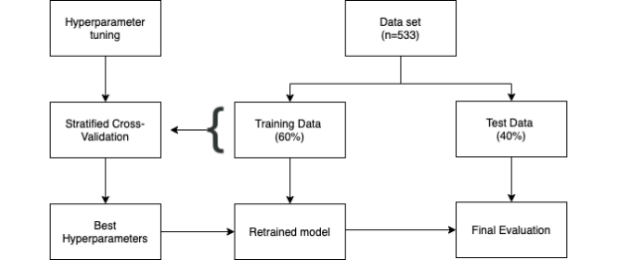
Overview of data set partitioning and model training.

Model development encompassed both the basic feature set and the engineered feature set. Because the study’s data set is composed out of labeled data with 2 distinct classes (normal observations and outliers), supervised learning can be applied in the form of a classification model. Because outliers are rare instances in the data, there is a class imbalance where the distribution between the normal observations and outliers is significantly skewed. To overcome the issue of class imbalance, a cost-sensitive learning technique was adopted, where the objective function of the classification algorithm is modified in order to weight the classification errors in a differential way for the normal and the less frequent class. This refers concretely to cost-sensitive logistic regression (CSLR), where a class weighting configuration is used to influence the amount of logistic regression coefficients that are updated during training. The weighting penalizes the model less for errors made on instances from the normal class, while maintaining a larger penalty for errors made on instances from the rare class. The result is a version of logistic regression that performs better on imbalanced classification tasks [17]. To avoid overfitting, a part of the available data set was held out as a test set during the data preprocessing phase (Figure 3). Moreover, in order to select the different hyperparameters related to our chosen models, a cross-validation strategy was adopted, more specifically in the form of stratified k-fold validation together with the hyperparameter optimization technique called grid search.

### Model Evaluation

To evaluate the effectiveness of the engineered features (Table 2), their performance was compared with that of the basic features (Table 1) and tested for significance. Therefore, 2 CSLRs were trained: one on the basic feature set and another on the engineered ones. All model performances were assessed on the same test set, which was composed of 40% of the total data and separated from the training set. The precision, recall, and the F1 score were chosen as performance evaluation metrics as these are best suited to evaluate data sets with class imbalance. Additionally, receiver operating characteristics (ROCs) and area under the curve (AUC) graphs were computed to characterize the performance of the models.

### Statistical Analysis

Statistical analysis was performed with the open-source python library Scikit-learn [22]. Wilcoxon signed-rank test was adopted to compare the performance of the CSLR models, with *P*<.05 considered significant.

## Results

### Descriptive Statistics, Model Training, and Cross-Validation

**Figure 4:**
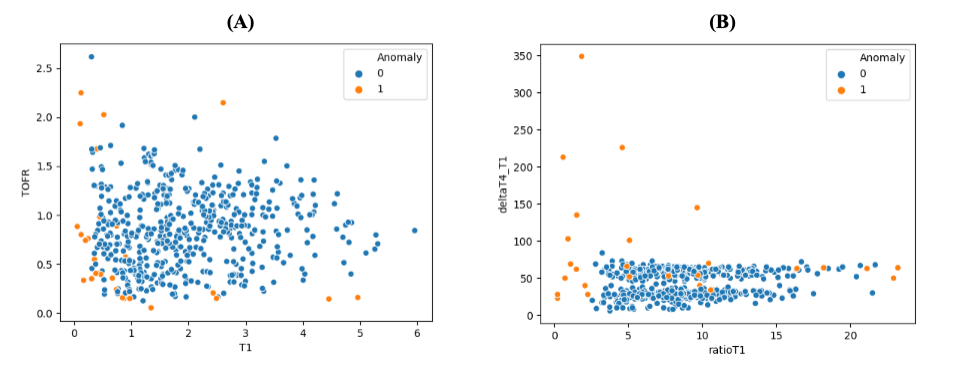
Panel A displays a scatter plot of train-of-four ratio (TOFR) and T1. Panel B displays a scatter plot of the features deltaT4_T1 and ratioT1. Scatter plot displays 1 as outlier, and 0 as a normal observation. TOFR in absolute units. DeltaT4_T1 in milliseconds. T1 in root-mean square angulation and acceleration.

The descriptive statistics of both feature sets are quantified in Table 3. The Python code related to hyperparameter optimization, training, and testing is presented in Multimedia Appendix 1. In contrast to the basic features, the engineered variables display a larger variation, as can be derived from their standard deviation. In fact, mainly the “delta” variables appear to be skewed. Figure 4 displays a scatter plot and a distribution plot of the basic features T1 and TOFR, and the engineered features ratioT1 and deltaT4_T1. While the outliers in the scatter plot of the basic features are more dispersed throughout the normal data, the outliers within the scatter plot of the engineered plots can more clearly be identified compared with the normal observations.

Concerning model training, the training data set (n=319) consisted out of 18 outliers, while the test data set (n=214) included 12 outliers. Both train and test data instances were chosen in a completely randomized manner by means of the train_test_split function of the scikit-learn library [22]. The division of data during cross-validation was performed solely on the segmented measurements (n=533). The split between train and test data has also been performed in a stratified way so as to guarantee the same class–imbalanced distribution of the entire data set. As for the stratified k-fold learning, 5 folds were chosen to split the training set, taking into consideration the size of the training set. In combination with cross validation, a grid search approach was employed to find the best hyperparameters for the L2 regularization coefficient and the appropriate class weights configuration for the imbalanced class distributions of our data set. All model training, cross-validation, and model evaluation were performed with the open-source library scikit-learn and the high-level programming language Python (version 3.8.2) [23].

**Table 3:** Descriptive statistics of basic features and engineered features.

Features	Mean (SD)	25%	50%	75%	Minimum	Maximum	Kurtosis	Skewness
T1	1.89 (1.13)	0.06	0.97	1.67	2.62	5.96	0.17	0.77
T2	1.82 (1.2)	0.02	0.8	1.63	2.58	6.03	0.09	0.78
T3	1.65 (1.25)	0.04	0.58	1.36	2.38	6.78	0.77	1.04
T4	1.6 (1.25)	0.05	0.56	1.26	2.34	6.3	0.62	1.05
TOFR^a^	0.84 (0.4)	0.05	0.53	0.82	1.1	2.62	0.48	0.52
AMG^b^_StdDev	0.44 (0.32)	0.05	0.18	0.36	0.61	2.01	2.54	1.31
AMG_Mean	0.27 (0.23)	0.04	0.11	0.2	0.35	1.5	5.91	2.06
deltaT2_T1	13.99 (6.55)	1	9	12	20	60	4.78	0.97
deltaT3_T2	14.44 (7.23)	2	9	13	20	80	20.16	2.47
deltaT4_T3	16.3 (18.95)	2	9	14	20	332	164.56	11.27
deltaT4_T1	44.73 (25.36)	6	28	48	60	349	46.29	4.61
ratioT1	8.55 (3.73)	0.22	5.96	7.95	10.41	23.21	1.39	0.94
ratioT2	7.57 (2.83)	0.04	5.72	7.23	9.09	20.19	1.27	0.73
ratioT3	6.52 (2.76)	0.09	4.47	6.16	8.17	18.46	1.46	0.92
ratioT4	6.36 (2.9)	0.19	4.3	6.05	8.15	18.47	1.03	0.74

^a^TOFR: train-of-four ratio.

^b^AMG: acceleromyography.

### Model Performance

**Figure 5:**
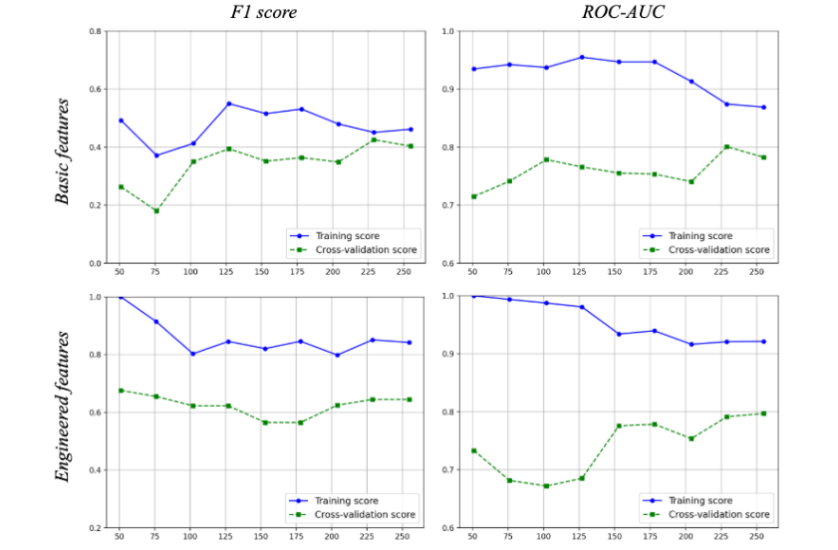
Learning curves of the cost-sensitive logistic regression models of the basic feature (above) set and the engineering feature set (below). Training and validation metrics are the F1-score and the ROC-AUC score (y-axis). X-axis represents the number of training instances.

Figure 5 presents the learning curves during model training and validation, for both the F1 score and ROC–AUC performance metrics. By plotting the model training and validation performances as functions of the training set size, high variance (ie, overfitting) or bias (ie, underfitting) can be assessed. While overall there seems to be a good bias–variance trade-off for both models, the CSLR based on the engineered features data set tends to overfit more than the model based on the basic features.

**Figure 6:**
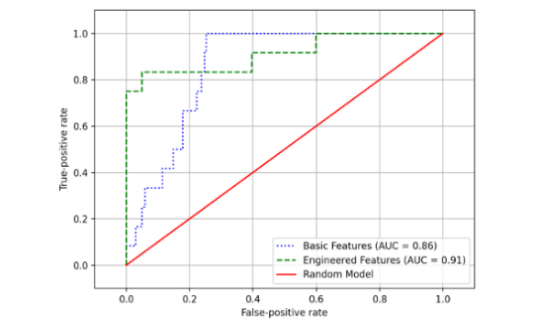
Receiver-operating characteristic curves displaying the ability of the algorithms to classify a train-of-four measurement as an outlier. AUC: area under the curve.

In Table 4, the F1 score, the ROC–AUC, precision, and recall for the CSLRs of the basic features and the engineered features are presented. Performance metrics are given for the training and test data sets. The CSLR model with the engineered features on the test data has an improved performance compared with the metrics of the training data set, indicating that the model has not been overfit. For the CSLR model of the basic features, we observe the opposite. The F1 score (95% CI) was 0.86 (0.48-0.97) for the CSLR model with the engineered features, which was significantly larger than the CSLR model with the basic features (0.29 [0.17-0.53]; *P*<.001). ROC curves and AUC curve results are visualized in Figure 6. The CSLR model with the engineered features has the highest AUC (95% CI) with a score of 0.91 (0.72-0.97), significantly larger than the CSLR model with the basic features (0.86 [0.63-0.93]; *P*<.001).

**Table 4:** Performance metrics of the training data set and the test data set.

Data sets			F1 score, mean (95% CI)	ROC^a^–AUC^b^, mean (95% CI)	Precision, mean (95% CI)	Recall, mean (95% CI)
**Training data set** **(n=319)**						
	Basic features		0.47 (0.24-0.63)	0.78 (0.63-0.82)	0.43 (0.18-0.68)	0.55 (0.33-0.71)
	Engineered features		0.65 (0.49-0.84)	0.80 (0.70-0.87)	1.00 (0.68-1.00)	0.50 (0.40-0.75)
**Test data set (n=214)**						
	Basic features		0.29 (0.17-0.53)	0.86 (0.63-0.93)	0.25 (0.10-0.52)	0.33 (0.29-0.98)
	Engineered features		0.86 (0.48-0.97)	0.91 (0.72-0.97)	1.00 (0.49-1.00)	0.75 (0.44-0.94)

^a^ROC: receiver operating characteristic.

^b^AUC: area under the curve.

## Discussion

### Principal Findings

The herein obtained results demonstrate that engineered TOF features outperform basic and common clinically employed neuromuscular monitoring endpoints for automated outlier identification of intraoperative TOF measurements. In the test data set, the CSLR of the engineered variables correctly identified 9 out of 12 measurements as outliers, compared with the CSLR of the basic features, which only correctly flagged one-third of the outliers. Moreover, the basic feature CSLR displayed a high degree of false positives, where 12 TOF measurements were incorrectly labeled as outliers, as opposed to 0 false positives on the CSLR model with the engineered features. While the authors recognize that the current models are sedimented on a limited data set and that further development is necessary in order to arrive at a clinically deployable outlier detection algorithm, the performance of the engineered feature algorithm is promising for a possible clinical application. Various research efforts [8,15,24] have highlighted that quantitative neuromuscular monitoring is still suboptimally and reluctantly adopted by practicing anesthesiologists, among others, due to a low perceived usefulness and reliability of monitoring devices. Even when effectively available on request, perceived unreliability due to artifactual recordings has been shown to be a prevalent barrier and a technical hindrance toward consistent monitoring adoption [15]. Nevertheless, the measurement error (artifact) incidence rates of standard neuromuscular monitors are unknown, and for that matter, so is an encompassing formal description and corresponding physiological correlations.

**Figure 7:**
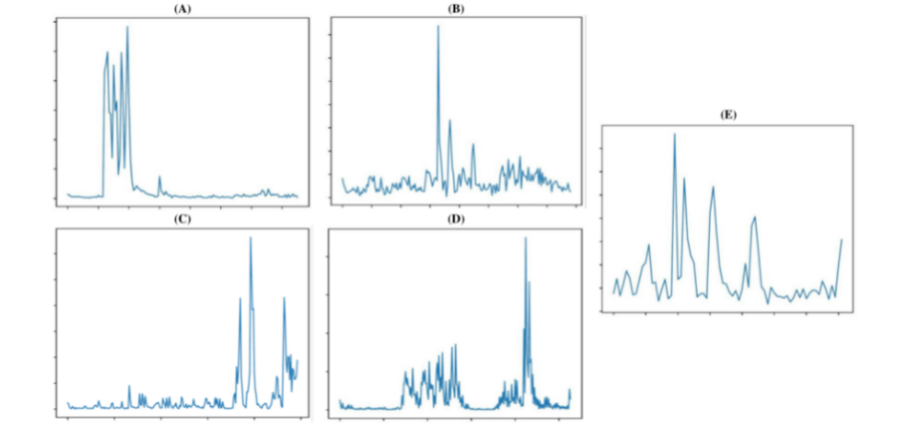
(A) Short 4-peak burst—rebound phenomenon TOF-like pattern after a single movement, short intertwitch distance; (B) T3 less than T2 and T4; (C) Almost equidistant oscillations; (D) Crescendo pattern; (E) Decrescendo but gross oscillations and variable interpeak distance. Some of the detected patterns have implications. y axis: muscle acceleration (m/s2), x axis: time (centiseconds). TOF: train-of-four.

Given the wide scope of anesthesia monitoring, the daily clinical relevance and eventual successful adoption of such an outlier analysis are certainly subject to debate. Nevertheless, the development of the CSLR models within this study is anchored on clinically grounded reported monitoring issues [7,8,15,25-29]. Figure 7 illustrates examples of flagged abnormal extraneural stimulation-induced moments. Although all have quantifiable and within-normality TOFR values, outliers are evident on closer inspection.

The anchoring of such outlier analysis to the clinical context of neuromuscular blockade monitoring is yet to be done practically and prospectively. Although the authors anticipate the present offline analysis will improve both neuromuscular monitoring adoption and clinical errors when embedded into anesthesia monitors, this can only be speculated upon at the present stage of development. It should additionally be reiterated that the bottleneck issue of undereducation is not tackled by the present developments. In fact, phenomena such as the failure of T1% to reach its baseline levels of around 100% during EMG-based neuromuscular monitoring are frequently observed in clinical practice and similarly trigger distrust among anesthesiologists [15]. The importance of human factors on the effective implementation of recommendation software has been highlighted by a recent randomized pilot trial, where a rather widely deployable predictive algorithm—the Hypotension Prediction Index (Edwards Lifesciences Corporation)—has been shown to fail to engage anesthesiologists [30].

As reinforced in the latest perioperative neuromuscular management consensus statement, educational efforts constitute an important part of modern anesthetic neuromuscular monitoring [7]. On the authors’ opinion, automated decision support software alone is expected to aid, but not solve or abolish, with the problem of suboptimal worldwide adoption of neuromuscular monitoring.

Considering the frequent and known artifactual biasing of kinetic data even with CE/FDA-labeled AMG/kinemyography/EMG neuromuscular monitoring devices [25-29], the added value of outlier analysis becomes especially relevant for reliability purposes. For instance, Liang et al [29] performed an ipsilateral comparison of AMG and EMG monitoring devices, concluding that AMG is less precise than EMG and overestimates the EMG TOFR by at least 0.15 units. A similar study performed by Kopman et al [28] found that AMG TOF values tend to overestimate the extent of EMG recovery, with a bias estimate of 0.125. However, both authors could not provide a cause to why these bias estimates between AMG and EMG devices were measured, stating that their lack of agreement cannot be explained by the imprecision of either device. Although outliers are not expected to explain such systematically reported intermethod precision differences when no technical issues are at hand, these do have the potential to compensate for small kinetic nuances such as overshoots, provided enough training data are available. In that sense, commonly applied AMG correction techniques such as normalization against baseline measurements could potentially be obviated. Nevertheless, based on the herein presented results, such potential is purely speculative.

### Study Limitations

- It can be argued that the data set that was collected and applied to develop the algorithm within this study is of a somewhat limited sample size. In order to address this limitation and avoid overfitting, we chose a cost-sensitive learning technique for logistic regression, adopted a cross-validation strategy together with the grid search optimization technique, and implemented regularization training, commonly used in machine learning.
- While the presented CSLR model with our engineered features is capable of detecting outliers in the process of AMG neuromuscular monitoring, there is no correlation with a possible cause. Hence, except for a measurement repetition, it remains unclear how clinicians would act upon the warning given by the algorithm.
- The data of this study were collected through an AMG-based smartphone app specifically aimed to monitor the TOF movement pattern and to calculate the corresponding TOFR. This app is undergoing further refinement to facilitate neuromuscular monitoring and to provide clinical intraoperative decision support. While the quality of the recorded measurements of the device has been previously assessed within a clinical trial [16], it is still possible that certain outliers are due to the nature of handheld devices themselves.
- We present a set of engineered features that have the potential for real-time detection of outliers within neuromuscular monitoring. Further analysis could reveal features that carry additional information for this purpose. Additionally, this study did not analyze (online) real-time streams of data to detect outliers.
- The algorithm developed depends on AMG neuromuscular monitoring devices. While these devices are still the most adopted quantitative neuromuscular devices in the domain of anesthesia [6], the developed algorithm does not tackle EMG-based devices [31].
- This study is not an intermethod validation study, but a precision increasing exercise that still warrants prospective intermethod comparison. This refers to its paired comparison with both AMG and EMG devices.

### Conclusion

This study demonstrates that a set of engineered features has the ability to detect outliers from an AMG neuromuscular device based on intraoperative measurements. The development of the model based on these features displayed promising results toward the creation of an outlier detection technique for neuromuscular monitoring. With further research and additional training, an outlier detection algorithm can potentially be implemented within an AMG neuromuscular monitoring device to scan TOF measurements for outliers automatically while not relying on active input from medical providers.

## Appendix

Multimedia Appendix 1 Jupyter Notebook including Python code of cost-sensitive logistic regression classifier.

## Abbreviations

AMG: acceleromyography
AUC: area under the curve
CSLR: cost-sensitive logistic regression
EMG: electromyography
ROC: receiver operator characteristics
TOF: train-of-four
TOFR: train-of-four ratio
